# Omega-3 Fatty Acids and Neurodegenerative Diseases: New Evidence in Clinical Trials

**DOI:** 10.3390/ijms20174256

**Published:** 2019-08-30

**Authors:** Rossella Avallone, Giovanni Vitale, Marco Bertolotti

**Affiliations:** 1 Department of Life Sciences, Modena and Reggio Emilia University, 41125 Modena, Italy; 2 Division of Geriatric Medicine, Department of Biomedical, Metabolic and Neural Sciences, and Center for Gerontological Evaluation and Research, Modena and Reggio Emilia University, 41126 Modena, Italy

**Keywords:** omega-3 polyunsaturated fatty acids, Parkinson’s disease, Alzheimer’s disease, clinical trials

## Abstract

A nutritional approach could be a promising strategy to prevent or slow the progression of neurodegenerative diseases such as Parkinson’s and Alzheimer’s disease, since there is no effective therapy for these diseases so far. The beneficial effects of omega-3 fatty acids are now well established by a plethora of studies through their involvement in multiple biochemical functions, including synthesis of anti-inflammatory mediators, cell membrane fluidity, intracellular signaling, and gene expression. This systematic review will consider epidemiological studies and clinical trials that assessed the impact of supplementation or dietary intake of omega-3 polyunsaturated fatty acids on neurodegenerative diseases such as Parkinson’s and Alzheimer’s diseases. Indeed, treatment with omega-3 fatty acids, being safe and well tolerated, represents a valuable and biologically plausible tool in the management of neurodegenerative diseases in their early stages.

## 1. Introduction

Several cerebral functions are determined by some nutrients, such as omega-3 polyunsaturated fatty acids (PUFAs), which are parts of the plasma membrane implicated in several processes, including increased synaptic development and functionality [1], effects on synaptic integrity and plasticity [2,3,4,5], contributing to neuroplasticity and subsequent enhancement of cognitive activity [6].

There is accumulating scientific evidence on the possible efficacy of PUFAs supplementation in neurodegenerative disorders [7,8], such as Parkinson’s (PD) and Alzheimer’s disease (AD) [9,10,11,12,13]. Although dietary recommendations are far from being a treatment for PD or AD, they may be able to alleviate some of the symptoms or slow the cognitive and physical decline.

The present study systematically reviews the effects of omega-3 polyunsaturated fatty acids’ supplementation on cognitive function in patients with Parkinson’s or Alzheimer’s disease.

The main classes of PUFAs belong to the omega-3 one, which comprises α-linolenic acid (ALA, 18:3 ω-3), eicosapentaenoic acid (EPA, 20:5 ω-3) and docosahexaenoic acid (DHA, 22:6 ω-3) and to the omega-6 one, which comprises linoleic acid (LA, 18:2 ω-6) and arachidonic acid (ARA, 20:4 ω-6) [14] (Table 1). DHA and ARA are the most important PUFAs in the brain [15]. In particular, DHA constitutes over 90% of the ω-3 PUFAs and 10–20% of total lipids in the brain [16]. It is mainly incorporated in phosphatidylethanolamine, phosphatidylserine and in smaller amounts in phosphatidylcholine [17] at synaptic terminals, mitochondria and endoplasmic reticula. Indeed, DHA is able to modulate cellular properties and physiological processes such as membrane fluidity, release of neurotransmitters, gene expression, myelination, neuroinflammation and neuronal growth [18,19].

**Figure 1:**
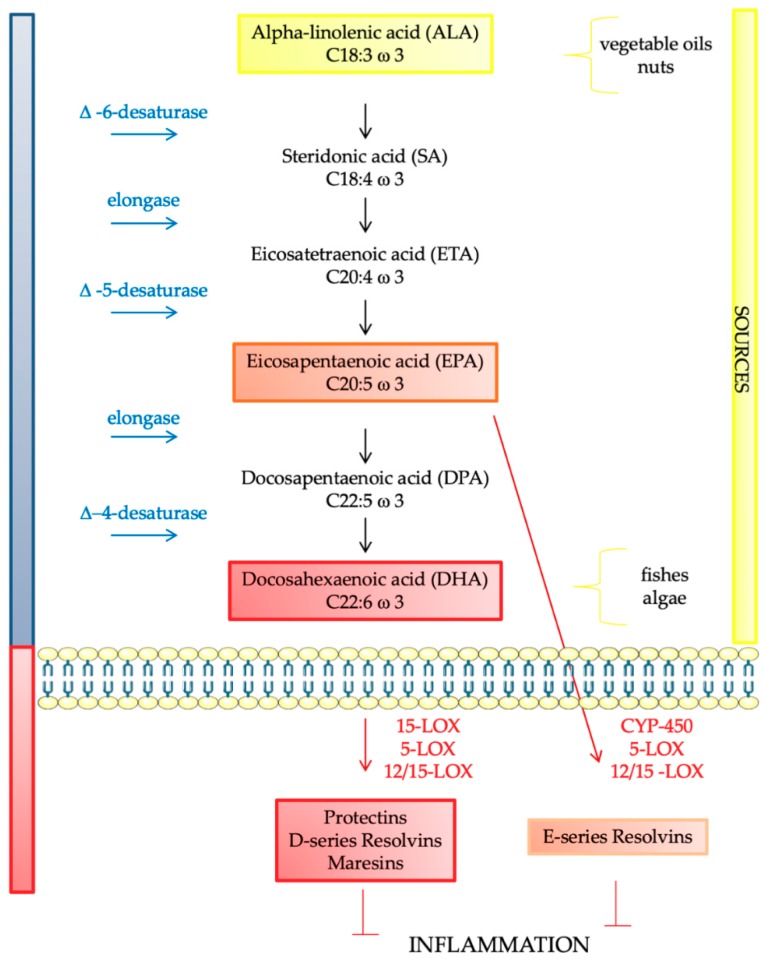
Endogenous synthesis of omega-3 polyunsaturated fatty acids and their involvement in inflammation. 15-LOX: 15-Lipoxygenase, 5-LOX: 5-Lipoxygenase, 12/15-LOX: 12/15 Lipoxygenase, CYP-450: Cytochrome P450.

DHA results from ALA, while ARA from LA by desaturation and elongation of the carbon chain [20] (Figure 1). Humans can synthesize saturated and monounsaturated fatty acids (MUFAs), but they are not able to synthesize ALA and LA due to the deficiency of the conversion enzyme ω-3-desaturase [21]. LA and ALA request the same conversion enzymes, consequently there is competitive inhibition between the two substrates. Delta-6-desaturase promotes the conversion of omega-3 fatty acids into omega-6 fatty acids. However, an increased LA intake may shift the balance towards the conversion of omega-6 PUFA thus inhibiting the conversion of ALA to DHA [22].

Esterified DHA in food, is released by the intestinal lipases in free unesterified form (DHA-FFA) in the small intestine and, after intestinal and hepatic metabolism, it can be found esterified in triglycerides and in phosphotidylcholine or as free DHA linked to low-density lipoprotein or albumin [15]. Endothelial lipases, fatty acid-binding proteins (FABP) and apolipoprotein E (ApoE) [23,24,25,26] dissociate the different forms in the blood-brain barrier (BBB) with both active and passive mechanisms [27]. The DHA, via FABP [24,25] and ApoE produced by astrocytes, [26] is transported within the central nervous system.

DHA is incorporated into membrane phospholipids mainly in the stereospecifically numbered-2 position through the action of coenzyme A [28]. However, through hydrolysis reactions catalyzed by phospholipase, DHA can be released from membrane phospholipids. Both synthesis and hydrolysis represent mechanisms aimed at responding to dynamic cellular events and challenges during development and aging [14].

DHA, EPA, and ARA are also important for the production of eicosanoids (prostaglandins, thromboxanes, leukotrienes) and, therefore, for their involvement in inflammation [20]. The phospholipase A2 enzymes (PLA2) hydrolyze the phospholipid by releasing fatty acids. As a result of an inflammatory stimulus involving specific cell activation, therefore, there is an increase in the levels of free fatty acids. Three types of PLA2 are mainly implicated in the release of bioactive lipids: the cytosolic calcium-dependent PLA2 (cPLA2), the cytosolic calcium-independent PLA2 (iPLA2) and the secretory PLA2 (sPLA2) [29]. Among them, cPLA2, shows a substrate specificity for phospholipids containing arachidonic acid (AA). cPLA2, however, can also hydrolyze phospholipids containing EPA, but the low abundance of this fatty acid allows cPLA2 to release AA in specific conditions [30]. Inflammatory modulation is regulated by prostaglandins, leukotrienes, and thromboxanes, which are metabolized by cyclooxygenase (COX) and 5-lipoxygenase (5-LOX) [31]. ARA is antecedent to the 2-series prostaglandins, as well as to thromboxanes and to 5-series leukotrienes [22]. Consequently, ARA has pro-inflammatory effects, while EPA has anti-inflammatory actions [22]. Furthermore, 5-LOX is responsible for the generation of anti-inflammatory eicosanoids such as the D-series resolvins, protectins and maresins, which are derived from DHA and the E-series resolvins from EPA [32,33] (Figure 1).

Human metabolic studies show a limited conversion of ALA to DHA, typically below 5% in adult males [34,35,36,37]. Women have a greater efficiency of conversion than men [38] and this may be important for fetal supply during pregnancy. Women demonstrated lower omega-3 fatty acid intake than men considering the same age categories [39]. Moreover, delta-6 desaturase activity decreases with age and undergoes lesser conversion, mainly in women. Therefore, to get the sufficient intake of EPA and DHA, especially in aging dietary supplements containing these preformed omega-3 are necessary. Indeed, the shift in modern diets towards reduced omega-3 PUFA intake increases omega-6 PUFA consumption and, if combined with less physical activity, has a detrimental impact on development and aging, especially with regard to cognitive function [14].

Current guidelines suggest an intake of EPA and DHA within the range of 250 to 500 mg [40]. As indicated by modern daily dietary, the consumption of omega-3 PUFAs is lower than necessary. DHA intakes, indeed, are closer to 100 mg per day, the optimal dietary omega-6 to omega-3 PUFA ratio has been determined in 2:1 or lesser, whereas the Western diet is usually established in the range of 10:1 to 25:1 [14].

The DHA daily dose necessary to induce significant positive results still requires further research. For example, a portion of 135 g of Atlantic salmon is necessary to reach 2 g/die of DHA [41] (Table 2). Thus, it is very difficult to achieve such high DHA consumption without integrations [7].

## 2. Parkinson’s Disease

Parkinson’s disease is a progressive neurodegenerative disorder characterized by loss of dopaminergic neurons in the substantia nigra, pars compacta. A pathological hallmark of the disease is also the presence of Lewy bodies, which are intracellular inclusions enriched in the protein α-synuclein.

The common symptoms include tremor, rigidity, bradykinesia and postural insecurity, with dementia and depression observed in the advanced stages of the disease [29]. In many instances, depression occurs before motor symptoms, which are typical expressions of Parkinson’s classic onset. In a recent study, it has also been shown that people suffering from depression are three-fold more predisposed to develop PD [44]. Depression, which affects a third of PD patients, combined with anxiety, apathy, and anhedonia further renders the PD outcomes even more complicated [29].

Although the etiology is currently unknown, there are a number of putative risk factors (e.g., exposure to environmental toxins) [45] and the pathogenic mechanisms include mitochondrial dysfunction, neuroinflammation, and oxidative stress [46]. However, numerous studies support that a diet rich in PUFAs or supplementation with food products containing EPA and DHA could alleviate some of the patients’ symptoms. The main scales used to evaluate PD symptoms are summarized in Table 3.

### 2.1. The Role of Omega-3 Polyunsaturated Fatty Acids in PD: Observational Studies

The first major prospective study concerning environmental, lifestyle, and physical precursors of clinical Parkinson’s disease is the Honolulu-Asia Aging Study [48], which started in 1965 and included a cohort of 8006 Japanese-American men, during a 30-year follow-up. Among the dietary factors showing an inverse association with PD, the polyunsaturated fats [48] were included (Table 4).

In The Rotterdam Study, the intakes of total fats, MUFAs and PUFAs were significantly associated with a lower risk of PD, by means of energy-adjusted intake of fat and fatty acids [49].

The association between dietary lifestyle and the risk of PD was evaluated in two most important studies: The Health Professionals Follow-Up Study (1986–2002) and the Nurses’ Health Study (1984–2000), including 131,368 men and women. Two dietary styles have been identified and compared: Prudent diet, characterized by high consumption of fruit, vegetables and fish and Western diet. It was demonstrated that the prudent diet significantly reduced PD risk, while the Western diet did not [50].

However, a case-control study, which examined the relationship between dietary intake of individual fatty acids and the risk of PD in Japan, including 249 cases within six years of onset of PD, demonstrated that, if the higher consumption of ARA and cholesterol could be related to an increased risk of PD, the intake of omega-3 polyunsaturated fatty acids was not [51].

In summary, we can conclude that prospective observational studies showed an association between a diet rich in polyunsaturated omega-3 fatty acids with a lower risk of PD.

### 2.2. The Role of Omega-3 Polyunsaturated Fatty Acids in PD: Randomized, Double-Blind, Placebo-Controlled Clinical Trials

Randomized, double-blind, placebo-controlled clinical trials involving PD are few for several reasons: poor patient adherence to diet therapy, duration of dietary treatment, control of clinical parameters and evaluation of these same parameters. When the pathology occurs, already 70% of neurons are compromised. Thus, thinking that only a dietary treatment can restore brain functions is really a utopia. However, dietary treatments with omega-3 fatty acids may have advantages in reducing inflammation and, consequently, depressive symptoms.

Indeed, treatment for six months with 800 mg/day DHA and 290 mg/day EPA from fish oil, demonstrated, in the DHA-treated patients, a reduction of 50% in the Hamilton rating scale for depression (HDRS) total score if compared with the placebo group which took corn oil. DHA integration reduced depressive symptoms [52]. However, treatment had no statistically significant effect on the rate of change on either unified Parkinson’s disease rating scale (UPDRS) or Hoehn-Yahr scale score [52] (Table 5).

Another double-blind, placebo-controlled study analyzed the effect of fish oil supplementation in parkinsonian patients with depression measured using Montgomery–Asberg rating scale (MADRS), the clinical global impressions scale (CGI) and Beck depression inventory (BDI) [44]. After three months, the supplementation with four capsules of omega-3 from fish oil (each capsule containing 180 mg EPA, 120 mg DHA and tocopherol) showed a significant decrease in MADRS and CGI-depression scores while there was no difference among treated and control groups in BDI [44]. Moreover, Parkinson’s symptoms, measured by Hoehn and Yahr scale, did not show significant variation during the three months of supplementation in all groups observed [44].

A randomized double-blind placebo-controlled clinical trial, conducted in 60 patients with PD, receiving either 1000 mg omega-3 fatty acids from flaxseed oil plus 400 IU vitamin E supplements or placebo for three months, showed that the dietetic supplementation in people with PD improved UPDRS, compared with the placebo [53].

The published papers give an important indication on the use of omega-3 supplements, especially for depression in PD. However, the number of patients recruited is small and also the types of supplements vary. It is widely demonstrated that the effective dose is 1 g/day of DHA. Animal or algal sources ensure a correct intake of DHA, while plant sources are often ineffective, since only 10% of ALA is metabolized to DHA. Despite this, the supplementation of omega-3 from linseed oil and vitamins E had favorable effects not only on UPDRS but also on high-sensitivity C-reactive protein (hs-CRP), total antioxidant capacity (TAC), glutathione and markers of insulin metabolism [53]. Furthermore, the three-to-six-month treatment is a relatively short period considering that we deal with dietary intervention in a pathology where the main symptoms are already evident [44,52,53].

## 3. Alzheimer’s Disease

Alzheimer’s Disease is a neurodegenerative syndrome that includes most cases of dementia, affecting over 35 million people all over the world. AD typical clinical features consist of cognitive impairment, memory loss, language disorders, rapid changes in mood and behavior, time- and space-disorientation, inhibition of the patients’ daily habits. The neurodegenerative process observed in AD is usually present in patients’ brain before the appearance of the first symptoms [54].

Neurofibrillary tangles, senile plaques, neuronal loss, and consequential brain atrophy represent the main features of AD.

Neurofibrillary tangles are formed by hyperphosphorylation and truncation of a protein, known as tau, which normally forms and stabilizes cytoskeleton by interaction with tubulin. As a result of the above-mentioned post-transcriptional modifications, tau protein can form toxic aggregates (i.e., neurofibrillary tangles) mainly situated in the hippocampus. Moreover, tau protein dysfunction produces cytoskeleton deconstruction as a result of microtubule disintegration, producing synaptic failure that produces a loss of communication and contributes to AD neurodegeneration.

Tau hyperphosphorylation also hinders mitochondrial transport since it is affected by interactions with microtubules, causing energy deficits in presynaptic areas that may result in a synaptic discontinuance.

**Figure 2:**
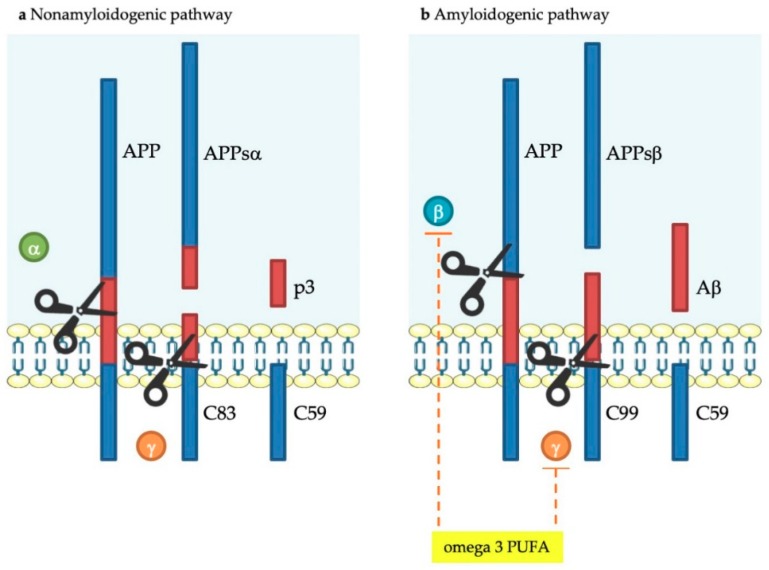
Amyloid precursor protein (APP) processing pathways. The non-amyloidogenic pathway (**a**) occurs upon sequential cleavage by α- and γ-secretases (non-pathological situation). The amyloidogenic pathway route (**b**) occurs when cleavage is carried out sequentially by β- and γ secretases (pathological situation). Letters α, β, and γ represent each type of secretase. APP: Amyloid precursor protein, APPsα: Soluble α-APP, APPsβ: Soluble β-APP. Omega-3 polyunsaturated fatty acids, proposed to inhibit APP processing, are shown in orange dashed lines.

The amyloid precursor protein (APP) degradation originates senile plaques constituted by extracellular deposits of β-amyloid peptide (Aβ) which induce inflammation and neuronal death. APP, a transmembrane protein present in neurons, can undergo cleavage by two different pathways: the amyloidogenic and the non-amyloidogenic ones, both mediated by secretases: β- and γ-secretases are involved in the first one, while α- and γ-secretases in the second one (Figure 2) [54]. In the non-amyloidogenic pathway, APP is sequentially cleaved by α-secretase and γ -secretase, generating truncation products: Aβ_17–40/42_ peptides (Figure 2). In the amyloidogenic pathway, APP is sequentially cleaved by β-secretase and γ-secretase, leading to whole-length Aβ peptides, responsible for the development of the plaques. While non-amyloidogenic pathway produces the amino-terminal fragment APPsα and the carboxy-terminal one C83, the amyloidogenic one produces APPsβ and C99. The activity of γ-secretase produces the APP intracellular domain (AICD), which takes part in the cellular signaling. Based on the point where γ-secretase generates the cut in the amyloidogenic pathway, the whole-length Aβ peptide would undergo different reductions: Aβ_1–40_ and Aβ_1–42_ are the main brain fragments (Figure 2) [54]. In the amyloidogenic processing, DHA decreased the β- and γ-secretase activity, while in the non-amyloidogenic processing it increased ADAM17 protein level, caused by a decreased protein degradation and an increased expression level [55]. Moreover, DHA lowers amyloidogenic processing by modifying both β- and γ-secretase activity with different mechanisms [55].

### 3.1. The Role of Omega-3 Polyunsaturated Fatty Acids in AD: Observational Studies

Prospective epidemiological studies performed in the Netherlands, USA, and France strongly supported a causal association between low fish and/or low DHA intake and AD. Most of the seven prospective studies published show that an increased intake of fish or omega-3 PUFA decreases the risk of AD (Table 6) [56].

The Rotterdam Study was the first to report that fish intake was inversely related to incidence of dementia, in particular to Alzheimer’s disease [57]. The data are confirmed by subsequent studies where the consumption of omega-3 [58] or fatty fish [59] or adherence to a diet rich in fruit, vegetables, fish, and oils rich in omega-3 [60,61,62,63], is associated with a reduction in AD risk. Subjects that had a mean DHA intake of 0.18 g/die (mean fish intake of three servings per week) have a lower relative risk of developing dementia if compared with subjects assuming lower DHA intake (0.15 g/die corresponding to mean fish intake of two servings per week).

Only a study published by Devore et al. [62] showed that differential consumption of fish (either 8.2 g/die or 29.6 g/die, defined as low or high medians) and omega-3 PUFAs, does not appear to be associated with long-term dementia risk. Results were also null for dietary intakes of EPA (0.03–0.08 d/die) and DHA (0.002–0.16 g/die) considering AD as the outcome of interest.

### 3.2. The Role of Omega-3 Polyunsaturated Fatty Acids in AD: Randomized, Double-Blind, Placebo-Controlled Clinical Trials

The first randomized clinical trial controlled by placebo (OmegAD Study) that evaluated omega-3 fatty acids’ impact in AD was published in 2006 [64] (Table 3 and Table 7).

A study by Freund-Levi et al. [64] evaluated omega-3 fatty acids’ supplementation in 204 subjects with mild to moderate AD. The active group received daily omega-3 fatty acids (1720 mg DHA and 600 mg EPA) while the placebo group received 4000 mg of corn oil (containing 2400 mg LA) for six months. Both groups received an additional six-month supplementation with omega-3 fatty acids. Medication for AD treatment was allowed. No significant difference was shown after a 6- or 12-month treatment between groups in Alzheimer’s disease assessment scale-cognitive subscale (ADAS-cog), mini-mental state examination (MMSE) and clinical dementia rating (CDR).

In a subgroup with very mild AD (MMSE > 27 and CDR 0.5–1), there was a significant reduction in decline rate between the intervention and placebo groups in the first six months.

In a second paper published in 2008, Freund-Levi et al. [65], using the same sample from 2006, showed that supplementation with omega-3 in patients with mild to moderate AD did not result in marked effects on neuropsychiatric symptoms except from possible positive effects on depression (assessed by MADRS) in non-APOEε4 carriers and agitation symptoms (assessed by Neuropsychiatric Inventory, NPI) in APOEε4 carriers. The omega-3 mechanism of action in the brain in relation to behavior is not fully elucidated. It has been shown in *in vitro* studies that a combination of EPA and DHA inhibits protein kinase C (PKC) activity [65]. Since mood stabilizers are known to inhibit PKC activity as well, PKC inhibition may represent a common mode of action for omega-3 in bipolar disorders. Other possible mechanisms could be that omega-3 fatty acids affect neurotransmitter levels and membrane fluidity also by decreasing production of pro-inflammatory eicosanoids that might be elevated in depression [65].

Chiu et al. [66] studied 46 subjects with mild to moderate AD or mild cognitive impairment (DSM-IV: MMSE 10–26, CDR-score 1–2). During six months, the intervention group received 720 mg/die DHA and 1080 mg/die EPA, while the placebo group received olive oil. Medication for AD treatment was not allowed. There was no significant statistical difference in MMSE, ADAS-cog, and HDRS between the two groups. The negative results of cognitive assessments support the previous studies by Freund-Levi et al. [64,65], and all of the studies showed there might be a positive effect of omega-3 fatty acids only in subgroups with mild cognitive deficits. A significant improvement was observed in clinician interview-based impression of change, plus carer interview (CIBIC-plus) in the intervention group compared to the placebo group. This might be explained considering the cognitive and behavioral aspects rather than the functional one. Omega-3 fatty acids may have been proposed to have beneficial effects on mood, although this is an unlikely explanation for these results because of the rigorous exclusion of people with significant depression and the absence of association with HDRS score. The relative progress of general clinical conditions might have been caused by improvement in cardiovascular or immunological systems induced by omega-3 [66].

In a subgroup, participants with mild cognitive impairment (MMSE >27 e CDR 0.5–1) but not with AD, showed a significant additional delay in ADAS-cog, decline compared to the placebo group.

Quinn et al. [67] assessed 402 subjects with mild to moderate AD. The intervention group received DHA 2000 mg/die from seaweed and the placebo group received corn or soy oil for 18 months. Medication for AD treatment was allowed. Supplementation with DHA compared with placebo did not slow the rate of cognitive and functional decline in patients with mild to moderate Alzheimer’s disease (no beneficial effect on the rate of change on MMSE, ADAS-cog, CDR, ADS-ADL, and NPI).

Sheltens et al. [68] assessed 225 subjects with mild AD. The intervention group received DHA 1700 mg/die and EPA 600 mg/die from a medical food named Souvenaid and the placebo group received a control drink for six months. Significant improvement in the delayed verbal recall task was noted in the supplemented group compared with control. ADAS-cog and other outcome scores (CIBIC-plus, NPI, ADCS-ADL) remained unchanged.

The same authors published a study [69] where the same above-mentioned population was divided into two subgroups: Patients with ‘low’ baseline ADAS-cog scores (<25.0) and patients with ‘high’ baseline ADAS-cog scores (≥25.0). Repeated measures models were used to determine the relationship between ADAS-cog score and intervention. Baseline ADAS-cog significantly influenced the effect of Souvenaid intervention on ADAS-cog outcome. A higher intake of medical food was also associated with greater cognitive benefit.

Based on these results, two double-blind, randomized controlled clinical trials were designed. The Souvenir II study examined the effect of longer treatment duration (six months) with Souvenaid, as compared with control product on memory performance in drug-naïve mild AD [70]. Neuropsychological test battery (NTB) memory domain increased in the active group.

Considering that ADAS-cog could be considerably modified in moderate AD and that Souvenaid had not been evaluated in patients with moderate AD already taking AD medications, a novel S-Connect study was planned. This double-blind, parallel, randomized, controlled clinical study, investigated the efficacy and tolerability of Souvenaid in 527 persons with mild to moderate AD, consuming constant doses of Souvenaid [71]. Cognitive performance evaluated by ADAS-cog, showed a decline over time in either placebo or active groups, indicating no significant difference between active groups themselves. Souvenaid drinking did not decelerate cognitive decline in patients treated for mild to moderate AD. Faxen-Irving et al., as a part of a previously published study on a DHA rich supplementation to subjects with AD [64], explored the effects of transthyretin on plasma and CSF. Since plasma transthyretin correlated with MMSE and inversely with ADAS-Cog, these authors suggest a potential mechanism for probable positive effects of omega-3 on cognition.

A study from Shinto et al. [73] investigated 39 subjects with probable AD in a randomized placebo-controlled pilot with three arms, one group receiving only omega-3 fatty acids (DHA 675 mg/die and EPA 975 mg/die), the second with the addition of alpha lipoic acid (600 mg/die), and the placebo group receiving soy oil. The intervention lasted 12 months and medication for AD was allowed. No differences were found in ADAS-cog and ADL between placebo and omega-3 fatty acids or between placebo and omega-3 fatty acids + alpha lipoic acid. In MMSE, the mean variation between the placebo group and the intervention group with only omega-3 fatty acids was not significant, whereas the difference between placebo and omega-3 fatty acids + alpha lipoic acid was significant. The mean IADL variation (Table 3) was significant between the placebo group and the omega-3 fatty acid group and between the placebo and the omega-3 fatty acids + alpha lipoic acid group.

In a secondary analysis of the Souvenir II study [74], results suggest that Souvenaid maintains the brain networks’ organization in patients with mild AD within six months, theoretically contrasting with the progressive network disruption over time in AD. These results strongly support the hypothesis that Souvenaid influences synaptic integrity and functioning.

Phillips et al. [75] assessed omega-3 fatty acids’ supplementation in 19 subjects with AD. The intervention group received daily omega-3 fatty acids in the dosages of 625 mg DHA and 600 mg EPA and the placebo group received olive oil for four months. The daily supplementation was associated with none or only negligible benefits on mood and cognition assessed by MMSE, the Hopkins verbal learning test-revised (HVLT-R), brief assessment schedule depression cards (BASDEC) and Bristol’s activities of daily living scale (BADLS).

Data obtained in the OmegAD study [65,80] were collected to examine the relationship of plasma omega-3 levels with cognitive scores (using ADAS-cog and the MMSE) [76]. The daily supplementation stabilizes the cognitive performance of AD subjects assessed by ADAS-cog and MMSE scores.

Also from the OmegAD study, a decrease was observed in resolvin D1 (RvD1) and lipoxin A_4_ (LXA_4_) production from peripheral blood mononuclear cells of AD patients who did not receive omega-3 supplementation but not in the cells of AD subjects under omega-3 intake [77].

Recent systematic meta-analysis did not show any significant benefits of omega-3 fatty acids supplementation in the treatment of mild to moderate AD, even if the treatment did not raise any substantial safety issues [13]. In fact, studies concerning the tolerability, safety, and effect size of Souvenaid demonstrated that the use of medical food for up to 12 months was well tolerated, with a favorable safety profile and high adherence of intake [78,81]. Moreover, the efficacy of omega-3 supplementation seems to be influenced by the baseline levels of plasma total homocysteine, suggesting that adequate B vitamin status is required to obtain beneficial effects of omega-3 on cognitive performance in moderate AD [79].

## 4. Materials and Methods

The authors searched PubMed, Web of Science, and Scopus articles using a combination of “omega-3 fatty acids,”, “Parkinson’s Disease, “Alzheimer’s Disease”, “clinical trials” as keywords. Inclusion criteria consisted of original intervention studies, controlled by placebo, that assessed omega-3 polyunsaturated fatty acids impact on cognitive function in humans with PD or AD, until May 2019, without restriction for the initial date of publication. We searched for interventions using omega-3 polyunsaturated fatty acids as dietary supplements or as increased dietary intake (such as fish or fish oils). First, we evaluated the titles and abstracts, then, we completed the reading of the full texts. Two reviewers independently performed the paper search, selection, and result extraction.

In order to favor reliability, data were collected independently in a table including the number of patients, population characteristics, type and dose of supplementation, exposure period, results and references. The authors prepared references using Zotero as bibliography software.

## 5. Conclusions

Neurodegenerative conditions, such as Parkinson’s disease and Alzheimer’s disease, represent a challenging issue in clinical medicine, and their burden is expected to increase dramatically in the forthcoming decades. At the present time, no etiological treatment is routinely available, and medical therapy is mainly symptomatic. The adoption of a nutritional approach would be highly recommendable.

Omega-3 fatty acids represent an interesting biological potential, in view of their anti-inflammatory and metabolic properties, in the management of these diseases.

Indeed, the evidence deriving from prospective observational studies is encouraging, both for Parkinson’s and Alzheimer’s disease. The adoption of a dietary regimen enriched with omega-3 fatty acids rather consistently associates with a reduced risk of either condition. On the other hand, randomized trials have provided conflicting results, and many of them have failed to document a definite protective effect. This was confirmed by most reviews and meta-analyses performed so far.

The inconsistency between observational and randomized studies is not unusual in clinical research, particularly when considering treatment with dietary supplements or integrators. A number of reasons may account for this finding. Firstly, in controlled trials, dietary supplementation is usually carried out over a relatively limited time span, compared with the life-long exposure of real-life observational studies. The different time course of the two approaches could play a relevant role. Observational studies may disclose the preventive effects of disease initiation, whereas in randomized trials involving patients already carrying a disease, the outcome more likely consists of a slowing of disease progression, or a reduction in disease-related complications. Distinct protective mechanisms are likely to take place. Furthermore, the variations in dietary patterns might reflect the adoption of a healthier lifestyle, in adjunct to the contribution provided by the single-nutrient supplementation. This was postulated, for instance, when investigating the protective effects of the Mediterranean diet on cognitive performances. In the present context, the intake of higher amounts of foods containing omega-3 fatty acids might be associated with a reduced intake of other nutrients, such as meat.

Finally, the possibility of different individual responses to dietary intervention must be considered. As mentioned in this review, the protective effects exerted by omega-3 fatty acids are likely to be modulated by patient-related factors, some of which may have a significant genetic component and may, therefore, be unmodifiable, and unpredictable with routine clinical and biochemical evaluation.

At any rate, treatment with omega-3 fatty acids was generally reported to be safe and well-tolerated. In our opinion, they may indeed represent a valuable and biologically plausible tool in the management of neurodegenerative diseases. Of course, supplementation needs to be a part of a global lifestyle intervention and has to take place in the early stages of the disease, when a benefit may be detected. Hopefully, in the near future the adoption of personalized treatment strategies, aimed to predict individual responses, will help to optimize the effectiveness of such intervention, in order to face the progressive rise of these devastating conditions.

## Figures and Tables

**Table 1:** Fatty acids’ categories.

Fatty Acids			
Saturated	Unsaturated		
	MUFA	PUFA	
	omega-9	omega-3	omega-6
stearic acid 18:0	oleic acid 18:1	ALA 18:3 EPA 20:5 DHA 22:6	LA 18:2 ARA 20:4

**Table 2:** Amount of α-linolenic acid (ALA), eicosapentaenoic acid (EPA), and docosahexaenoic acid (DHA).

Food	ALA g/Portion	EPA g/Portion	DHA g/Portion
Baked beans, canned, vegetarian	0.07		
Black walnuts	0.76		
Bread, whole wheat	0.04		
Canola oil	1.28		
Chia seeds	5.06		
Chicken, breast, roasted,		0.01	0.02
Cod, Pacific, cooked *		0.04	0.10
Edamame, frozen, prepared	0.28		
Egg, cooked			0.03
English walnuts	2.57		
Flaxseed oil	7.26		
Flaxseed, whole	2.35		
Ground beef, 85% lean, cooked **	0.04		
Herring, Atlantic, cooked *		0.77	0.94
Kidney beans, canned	0.10		
Lobster, cooked *	0.04	0.10	0.07
Mackerel, Atlantic, cooked *		0.43	0.59
Mayonnaise	0.74		
Milk, low-fat (1%)	0.01		
Oysters, eastern, wild, cooked	0.14	0.30	0.23
Refried beans, canned, vegetarian	0.21		
Salmon, Atlantic, farmed, cooked		0.59	1.24
Salmon, Atlantic, wild, cooked		0.35	1.22
Salmon, pink, canned, drained *	0.04	0.28	0.63
Sardines, canned in tomato sauce, drained *		0.45	0.74
Scallops, cooked *		0.06	0.09
Sea bass, cooked *		0.18	0.47
Shrimp, cooked *		0.12	0.12
Soybean oil	0.92		
Tilapia, cooked *	0.04		0.11
Trout, rainbow, wild, cooked		0.40	0.44
Tuna, light, canned in water, drained *		0.02	0.17
Tuna, yellowfin, cooked *		0.01	0.09

* Except as noted, the USDA database does not specify whether fish are farmed or wild-caught. ** The United States Department of Agriculture Food Composition Databases does not specify whether beef is grass-fed or grain-fed. Data from Office of Dietary Supplements, National Institute of Health (NIH) [42,43].

**Table 3:** Summary of main scales used for assessment of Parkinson’s and Alzheimer’s Disease ([47] with modifications).

Main Scales	Description
Activities of Daily Living ADCS-ADL, ADCS-IADL	It measures the functional ability to perform activities of daily life. ADL assess basic living skills such as bathing and eating, whereas Instrumental activities of daily living (IADL) measures more complex tasks such as using the telephone or preparing meals. A higher ADL or IADL score indicates a worsening functionality.
Alzheimer’s Disease Assessment Scale-Cognitive Subscale (ADAS-Cog)	It is a sensitive and reliable method for the assessment of cognitive function in dementia. It consists of a psychometric scale of 11 items, and scores range from 0 (no impairment) to 70 (very severe impairment).
Beck Depression Inventory (BDI)	It is a 21-question multiple-choice self-report inventory, one of the most widely used psychometric tests for measuring the severity of depression.
Brief Assessment Schedule Depression Cards (BASDEC)	It is a brief test for screening depression, requiring minimal training to administer.
Bristol’s Activities of Daily Living Scale (BADLS)	It is specifically designed for individuals with mild dementia living in the community for completion by caregivers.
Clinical Dementia Rating (CDR)	It is a global measure that assesses memory, orientation, judgment, and other features. Is based on caregiver interview. Classifies dementia into questionable, mild, moderate, and severe.
Clinical Global Impression Scale (CGI)	It measures symptom severity, treatment response, and the efficacy of treatments in treatment studies of patients with mental disorders.
Clinician Interview-Based Impression of Change, plus carer interview (CIBIC-Plus)	It is a global measure capable of detecting changes in cognition, functionality, and behavior, thus assessing dementia’s severity and progression. Requires separate interviews with patients and caregivers.
Diagnostic and Statistical Manual of Mental Disorders, fourth edition (DSM-IV)	It is the handbook used by health care professionals in the United States and much of the world as the authoritative guide to the diagnosis of mental disorders.
Hamilton Depression Rating Scale (HDRS)	Is the most widely used clinician-administered depression assessment scale. The original version contains 17 items pertaining to symptoms of depression experienced over the past week.
Hoehn and Yahr scale	It is a commonly used system for describing how the symptoms of PD progress.
Hopkins Verbal Learning Test–Revised (HVLT-R)	It is a brief verbal learning and memory test ideal in situations calling for repeated neuropsychological examinations.
Mini-Mental State Examination (MMSE)	It evaluates cognitive function in the areas of orientation, memory, attention, calculation, language, and visual construction. It is widely translated and used in clinical practice. Patients score between 0 and 30 points, and cutoffs of 23/24 have typically been used to show significant cognitive impairment.
Montgomery–Åsberg Depression Rating Scale (MADRS)	It is a ten-item diagnostic questionnaire which psychiatrists use to measure the severity of depressive episodes in patients with mood disorders.
Neuropsychiatric Inventory (NPI)	It assesses dementia-related behavioral symptoms. The NPI originally examined 10 sub-domains of behavioral functioning: Delusions, hallucinations, agitation/aggression, dysphoria, anxiety, euphoria, apathy, disinhibition, irritability/lability, and aberrant motor activity.
Neuropsychological test battery (NTB)	This scale assesses changes in cognitive function and is seen as a promising method for mild AD. NTB has shown to be able to detect changes in memory performance.
Unified Parkinson’s Disease Rating Scale (UPDRS)	It evaluates motor impairment and disability of patients with PD.

**Table 4:** Prospective observational studies assessing the impact of omega-3 fatty acids supplementation in Parkinson’s disease PD patients.

N° Patients	Population Characteristic	Type and Dose Supplementation	Exposure Period	Results	References
8006	PD Honolulu-Asia Aging Study	Food frequency questionnaire	30 years	Omega-3 PUFAs appeared protective.	[48]
5289	PD Rotterdam Study The Netherlands	Semiquantitative food frequency questionnaire	6 years	Intakes of omega-3 PUFAs were significantly associated with a lower risk of PD.	[49]
131,368	PD Health Professionals Follow-Up Study and the Nurses’ Health Study USA	Semiquantitative food frequency questionnaire	16 years	High intakes of fruit, vegetables, whole grains, legumes, poultry, and fish were associated with a lower risk of PD.	[50]
249	PD Japan	Self-administered diet history questionnaire	6 years	Consumption of omega-3 PUFA, ALA, EPA, DHA was not associated with PD.	[51]

**Table 5:** Clinical trials assessing the impact of omega-3 fatty acids supplementation in PD patients.

N° Patients	Population Characteristics	Type and Dose Supplementation	Exposure Period	Results	References
24	PD Italy	800 mg/die DHA + 290 mg/die EPA from fish oil Placebo: Corn oil	6 months	Treatment had no statistically significant effect on the rate of change on either UPDRS or Hoehn-Yahr Scale score. In DHA-treated patients, the HDRS score was reduced by at least 50%.	[52]
31	PD and Major Depression (DSM-IV) Brazil	480 mg/die DHA + 720 mg/die EPA from fish oil + tocopherol Placebo: Mineral oil	3 months	Treatment had no statistically significant effect on the rate of change on Hoehn-Yahr Scale score, but there was a significant decrease in MADRS and CGI scores.	[44]
60	PD Iran	1000 mg omega-3 fatty acids from flaxseed oil + 400 IU vitamin E Placebo: Not specified	3 months	Treatment had favorable effects on UPDRS score.	[53]

**Table 6:** Prospective observational studies assessing the impact of omega-3 fatty acids supplementation in AD patients.

N° Patients	Population Characteristic	Type and Dose Supplementation	Exposure Period	Results	References
5386	AD 37 Rotterdam Study The Netherlands	Semiquantitative food frequency questionnaire	2.1 years	Fish consumption, an important source of omega-3 PUFA, was inversely related to incident dementia, in particular to Alzheimer’s disease.	[57]
815	AD 131 Chicago Health and Aging Project USA	Food frequency questionnaire	3.9 years	Dietary intake of omega-3 PUFA and weakly consumption of fish may reduce the risk of Alzheimer’s disease.	[58]
2233	AD 190 Cardiovascular Health Cognition Study (CHCS) USA	Food frequency questionnaire	5.4 years	Consumption of fatty fish more than twice per week was associated with a reduction in the risk of Alzheimer’s disease by 41%.	[59]
488	AD not reported The Framingham Heart Study USA	Semiquantitative food frequency questionnaire	9.1 years	Plasma DHA level was associated with a significant 47% reduction in the risk of developing all-cause dementia.	[60]
8085	AD 183 Three-City cohort study France	Food frequency questionnaire	3.48 years	Frequent consumption of fruits and vegetables, fish, and omega-3 rich oils may decrease the risk of dementia and Alzheimer’s disease, especially among ApoE ε4 noncarriers.	[61]
5395	Rotterdam Study The Netherlands	Semiquantitative food frequency questionnaire	9.6 years	In the cohort with moderate consumption of fish and omega-3 PUFAs these dietary factors did not appear to be associated with long-term dementia risk	[62]
923	AD Rush Memory and Aging Project USA	Semiquantitative food frequency questionnaire	4.5 years	High adherence to all three diets may reduce AD risk.	[63]

**Table 7:** Clinical trials assessing the impact of omega-3 fatty acids supplementation in AD patients.

N° Patients	Population Characteristic	Type and Dose Supplementation	Exposure Period	Results	References
204	AD (DSM-IV) MMSE 15–30 OmegAD Study	1720 mg/die DHA+ 600 mg/die EPA Placebo: 4000 mg corn oil Both groups: + 16 mg/die vitamin E	12 months	There was no significant statistical difference after 6- or 12-month treatment between groups in MMSE, ADAS-cog, CDR. A subgroup with very mild cognitive dysfunction showed a reduction in decline rate.	[64]
204	AD (DSM-IV) MMSE 15–30 OmegAD Study	1720 mg/die DHA+ 600 mg/die EPA Placebo: 4000 mg corn oil Both groups: + 16 mg vitamin E	12 months	Supplementation with omega-3 did not result in marked effects on neuropsychiatric symptoms except for possible positive effects on depressive symptoms (assessed by MADRS) in non-APOEε4 carriers and agitation symptoms (assessed by NPI) in APOEε4 carriers.	[65]
46	AD AD (DSM-IV) MMSE 10–26 CDR-score 1–2	720 mg/die DHA+ 1080 mg/die EPA Placebo: Olive oil Both groups: + 1.2 mg hydroquinone + 12 mg tocopherols	6 months	The treated group did not show an improvement in cognitive symptoms measured by MMSE, ADAS-cog, HDR but a relative improvement in CIBIC-plus score. In a subgroup with subjects with mild cognitive impairment (MMSE >27 e CDR 0.5–1) there was an improvement in ADAS-cog.	[66]
402	AD MMSE 14–26 Alzheimer’s Disease Cooperative Study (ADCS) DHA Supplementation Trial USA	2000 mg/die DHA from seaweed Placebo: Corn or soy oil	18 months	Supplementation with DHA compared with placebo did not slow the rate of cognitive and functional decline in patients with mild to moderate Alzheimer’s disease assessed by MMSE, ADAS-cog, CDR, ADS-ADL, NPI.	[67]
225	AD Souvenir I Study	1700 mg/die DHA+ 600 mg/die EPA (Souvenaid) Placebo: Control drink	6 months	Supplementation with omega -3 improved delayed verbal recall. However, ADAS-cog, CIBIC-plus, NPI, ADCS-ADL, ADSC-ADL were unchanged.	[68]
225	AD Souvenir I Study	1700 mg/die DHA+ 600 mg/die EPA (Souvenaid) Placebo: Control drink	6 months	Souvenaid had a positive result on ADAS-cog outcome. A higher intake of Souvenaid was also associated with greater cognitive benefit.	[69]
238	AD Souvenir II Study	1200 mg/die DHA+ 300 mg/die EPA (Souvenaid) Placebo: Control products	6 months	In the active group, the NTB memory domain increased.	[70]
527	AD MMSE 14 – 24 Connect Study	1200 mg/die DHA+ 300 mg/die EPA (Souvenaid) Placebo: Control products	6 months	Cognitive performance, as assessed by ADAS-cog, showed a decline over time in both control and active study groups, with no significant difference between study groups. Add-on intake of Souvenaid did not slow cognitive decline in persons treated for mild-to-moderate AD.	[71]
174	AD mild to moderate OmegAD Study	1720 mg/die DHA+ 600 mg /die EPA Placebo: 4000 mg corn oil	12 months	Plasma transthyretin positively correlated with MMSE and inversely with ADAS-Cog, suggesting a potential mechanism for probable positive effects of omega-3 on cognition.	[72]
39	AD MMSE 15–26 CDR 0.5–1.0 Not depressed (CESD <4.0)	675 mg/die DHA+ 975 mg /die EPA Group omega-3 plus alpha lipoic acid (LA): 675 mg/die DHA+ 975 mg/die EPA+ 600 mg/die LA Placebo: Soy oil	12 months	Active groups were no different from the placebo group in ADAS-cog, ADL. Omega-3 + LA group showed less decline assessed by MMSE. IADL differences between placebo e omega-3 and between placebo e omega-3 + LA groups were observed.	[73]
179	AD mild Souvenir II Study	1700 mg/die DHA+ 6 mg/die EPA (Souvenaid) Placebo: Control drink	6 months	The administration contributed to the maintenance of the organization of brain networks in mild AD patients.	[74]
19	AD MMSE 16–30	625 mg/die DHA+ 600 mg/die EPA Placebo: Olive oil Both groups: + 20 mg mixed tocopherols	4 months	The daily supplementation was associated with none or only negligible benefits on mood and cognition, assessed by MMSE, HVLT-R, BASDEC, BADLS.	[75]
204	AD OmegAD Study	1720 mg/die DHA+ 600 mg/die EPA Placebo: 4000 mg corn oil Both groups: + 16 mg vitamin E	6 months	The daily supplementation stabilized the cognitive performance of AD subjects, assessed by ADAS-cog and MMSE scores.	[76]
204	AD OmegAD Study	1720 mg/die DHA+ 600 mg/die EPA Placebo: 4000 mg corn oil Both groups: + 16 mg vitamin E	6 months	A decrease was observed in RvD1 and LXA4 production from peripheral blood mononuclear cells of AD patients who did not receive omega-3 but not in cells of AD subjects under omega-3 intake.	[77]
201	AD Open label extension study (OLE) Souvenir II MMSE ≥ 20	1200 mg/die DHA+ 300 mg/die EPA (Souvenaid) Placebo: Control drink	6 months	The intake of Souvenaid was well tolerated with a favorable safety profile. The adherence to Souvenaid was very high reflecting its good tolerability and ease of use.	[78]
171	AD OmegAD Study	1720 mg/die DHA+ 600 mg/die EPA Placebo: 1 g corn oil Both groups: + 16 mg vitamin E	6 months	The effect of omega-3 supplementation on MMSE and CDR appeared to be influenced by homocysteine plasma levels.	[79]

## Abbreviations

5-LOX	5-lipoxygenase
AD	Alzheimer’s disease
ADAS-Cog	Alzheimer’s disease assessment scale–cognitive subscale
ADCS	Activities of daily living scales
ALA	α linolenic acid
ApoE	apolipoprotein E
APP	amyloid precursor protein
ARA	arachidonic acid
Aβ	amyloid beta peptide
BADLS	Bristol’s activities of daily living scale
BASDEC	brief assessment schedule depression cards
BBB	blood–brain barrier
BDI	Beck depression inventory scale
CDR	clinical dementia rating scale
CGI	clinical global impression scale
CIBIC-Plus	clinician interview-based impression of change, plus carer interview
COX	cyclooxygenase
cPLA2	cytosolic calcium-dependent phospholipase A2
CSF	cerebrospinal fluid
DHA	docosahexaenoic acid
DHA-FFA	free nonesterified form docosahexaenoic acid
DSM-IV	Diagnostic and Statistical Manual of Mental Disorders, fourth edition
EPA	eicosapentaenoic acid
FABPs	fatty acid binding proteins
HDRS	Hamilton depression rating scale
hs-CRP	C-reactive protein
HVLT-R	Hopkins verbal learning test–revised
iPLA2	cytosolic calcium-independent phospholipase A2
LA	linoleic acid
LXA_4_	lipoxin A_4_
MADRS	Montgomery–Åsberg depression rating scale
MMSE	mini-mental state examination
MUFAs	monounsaturated fatty acids
NPI	Neuropsychiatric Inventory
NTB	neuropsychological test battery
PD	Parkinson’s disease
PKC	protein kinase C
PLA2	phospholipase A2
PUFAs	omega-3 polyunsaturated fatty acids
sPLA2	secretory phospholipase A2
RvD1	resolvin D1
TAC	total antioxidant capacity
UPDRS	unified Parkinson’s disease rating scale
